# Mycelium Growth and Development of *Psilocybe* spp. Mother Cultures on Agar-Based Media

**DOI:** 10.3390/jof11060450

**Published:** 2025-06-13

**Authors:** Marco Pepe, Mohsen Hesami, Livia Fleishmann, Tom Hsiang, Andrew Maxwell Phineas Jones

**Affiliations:** 1 Department of Plant Agriculture, University of Guelph, Guelph, ON N1G 2W1, Canada; pepem@uoguelph.ca (M.P.); mhesami@uoguelph.ca (M.H.); 2 School of Engineering, University of Guelph, Guelph, ON N1G 2W1, Canada; fleischl@uoguelph.ca; 3 School of Environmental Sciences, University of Guelph, Guelph, ON N1G 2W1, Canada; thsiang@uoguelph.ca

**Keywords:** *Psilocybe*, psilocybin, mushroom production, mycelium, mycology

## Abstract

The resurgence of interest in the therapeutic potential of psilocybin-producing mushrooms has recently led to numerous research and commercialization efforts. Due to its ease of cultivation and high potency, *Psilocybe* is the primary genus of interest, and there is a growing need to standardize maintenance, proliferation, and cultivation techniques for efficient and consistent production. The investigation of mycelial growth and development on agar-based media is of principal importance to regulate and optimize mycelium growth and preservation protocols for subsequent fruiting body development. The current investigation is the first to examine the mycelial growth and morphology of four *Psilocybe* genotypes cultured on different agar-based media. The results from this simple set of experiments provides the foundation for future optimization studies. Ultimately, the information presented can be used to develop genotype-specific mycelial growth and development practices that will shape the future of psychedelic mushroom production for clinical and industrial applications.

## 1. Introduction

Recently, the therapeutic potential of psychedelic substances has revitalized interest in the research and commercialization of psilocybin-producing mushrooms [1,2,3]. The clinical implications of psilocybin-assisted therapy include but are not limited to substance use disorder, depression, and anxiety. There is particular interest in *Psilocybe* spp. isolates since they are typically easy to cultivate and often associated with high potency [1]. Growing interest and awareness surrounding these psychoactive natural products has contributed to certain countries easing restrictions on the cultivation, study, and therapeutic application of these fungi [1,4]. Since synthetic psilocybin can be produced in accordance with the regulations of good manufacturing practices (GMPs), they currently set the standard for clinical research. However, the use of synthetically derived, isolated mushroom compounds does not fully represent the effects of the chemically complex mushrooms that are often used in practice [1]. This discrepancy establishes a need to standardize effective techniques for the maintenance and proliferation of mycelia and the production of *Psilocybe* spp. fruiting bodies.

The culturing of mycelia on semi-solid media is an essential practice in mycology. The growth and development of different fungal genotypes can be highly inconsistent across different media [5]. The ability to support the growth of mycelia is largely associated with the nutritional and organic content of the media [6,7], which can differ depending on the source material. Of the semi-solid media available, potato dextrose agar (PDA) and malt extract agar (MEA) are among the most common in scientific research and cultivation [8,9,10]. Different substrates can induce variability related to growth rate and developmental features, such as hyphal density [11]. Additionally, the preparation of agar-based substrates from natural raw materials can be tedious and time-consuming, leading to the commercialization of pre-made, powdered formulations [9]. While these options are convenient, they can be more expensive [12]. Of equal importance, media made from natural products such as potatoes and malt are inherently variable, and quality can be influenced by many factors, including the source of raw ingredients and dextrose quality, which can impact formulation accuracy.

The variability of starting material and preparation methods can affect the growth rates and developmental properties of mycelia. For instance, commercial PDA can result in differential metabolite production [13,14], pigmentation [9,14], or growth rates [15] compared to PDA prepared fresh with locally sourced potatoes. While powdered alternatives can improve consistency and replicability related to mycelium proliferation, the use of PDA from different suppliers can lead to alterations in mycelial properties [13]. The associated variability could be due to mineral elements [9,16,17], vitamins, amino acids, or lipids that may be available in the raw ingredients but absent in the commercially prepared products. Although these components can potentially be introduced by adding peptone, yeast, or beef extracts [14,18], their inclusion increases the associated production costs and can potentially result in the occurrence of superfluous organic compounds. This source of variability complicates the standardization of effective practices for mycelial growth, which has a cascading effect on the cultivation system as a whole. Thus, it is important to understand the impacts of media sources and preparation methods on mycelial growth and development.

The push to standardize practices for culturing *Psilocybe* spp. has fueled the current investigation with the aim to track the mycelial growth rates and morphologies of four *Psilocybe* genotypes on semi-solid media. The select genotypes were cultured on several preparations of potato dextrose agar (PDA), malt extract agar (MEA), Murashige and Skoog basal salt formulation (MS), and water agar (WA). The various preparations of PDA included versions freshly made with locally sourced, unpeeled potatoes (PDA P), peeled potatoes (PDA), and a commercially available potato dextrose powder (PDA R). In line with the objective, this set of experiments revealed significant differences in growth among the media and highlighted the diverse responses among genotypes. Ultimately, the information reveals the importance of mother culture media composition and the need to optimize for different genotypes. This will allow the implementation of good agricultural and collection practices (GACPs) that are compliant for the production of psychedelic mushrooms.

## 2. Materials and Methods

### 2.1. Fungal Material

The research conducted was separated into two series of experiments, both looking at the growth and morphology of the same four genotypes of *Psilocybe* mushrooms. The genotypes included *P. cubensis* “Albino Penis Envy” (APE), *P. cubensis* “B+” (B+), *P. cubensis* “Jedi Mind Fuck” (JMF), and *P. allenii* (PA). These were chosen based on their differences in vigour, ease of cultivation, and potential potency, as documented throughout the user-based literature, also known as the “grey literature” (tripsitter.com/magic-mushrooms/strains/ accessed on 2 March 2 2025). Spores of APE and JMF were supplied from an anonymous donor by the alias Tamijed (Chicago, IL, USA), B+ spores were donated by Marco Pepe (Guelph, ON, Canada), and PA was donated by Richard Philbrook (Martinez, CA, USA).

The *P. cubensis* genotypes selected are commonly cultivated and traded. To verify that the isolates used are the correct genotypes, fruiting bodies were produced for morphological assessment. It is with extremely high probability that the *P. cubesis* mycelia used are isolates of the genotypes mentioned above. As for the *P. allenii* isolate, the internal transcribed spacer (ITS) region was sequenced via GENEWIZ (South Plainfield, NJ, USA), and the ITS sequence showed 100% alignment with *P. allenii*, using BLASTn (NCBI).

Spores were germinated in sterilized distilled water, transferred to agar-based semi-solid substrates, and sub-cultured for several months to ensure pure cultures before both experimental series. Mycelium for both experimental series was grown for one month in the dark, at room temperature. The parental mycelium used for Experimental Series 1 was originally grown on ¼-strength potato dextrose agar and then transferred to experimental treatments. The parental mycelium used for Experimental Series 2 was initially grown on water agar and then transferred to experimental treatments (see following section for additional details relating to each media treatment).

### 2.2. Experimental Treatments and Media Preparation

Media treatments for Experimental Series 1 included potato dextrose agar made with peeled potatoes (PDA), malt extract agar (MEA), Murashige and Skoog basal salt formulation (MS), and water agar (WA). Media treatments for Experimental Series 2 included PDA, potato dextrose agar made with unpeeled potatoes (PDA P), powdered potato dextrose broth mixed with agar (PDA R), and WA. For these experimental series, WA was chosen to represent a pseudo-control. Due to the lack of carbohydrates and nutrients, WA allows the rapid colonization of sparse mycelium colonies. Thus, media treatments were compared amongst themselves and to WA to assess growth and morphology. Further details relating to media recipes are depicted in Table 1. For PDA and PDA R, potatoes were rough-cut into 2 cm^3^ morsels and added to boiling water for 30 min. Potato broth was then filtered out using a cheese cloth. Media components were then mixed and dissolved in distilled water. In all cases, the solutions’ pHs were adjusted to 5.6 ± 2 using KOH and HCl before the agar was added at 20 g/L. Media treatments were then autoclaved and dispensed into 85 mm diameter Petri dishes, each containing 30 mL of media. Additional details related to media preparation and media ingredients are presented in Table 1.

“Your Fresh Market” brand “Red Sangre” potatoes used for PDA and PDA P treatments were obtained from Walmart (Guelph, ON, Canada). Agar, dextrose, and MS basal salt formulation were obtained from PhytoTech Labs Inc. (Lenexa, KS, USA). BD Difco brand potato dextrose broth and Oxoid brand malt extract broth were used for PDA R and MEA treatments, respectively (Fisher Scientific, Missisauga, ON, Canada).

**Figure 1:**
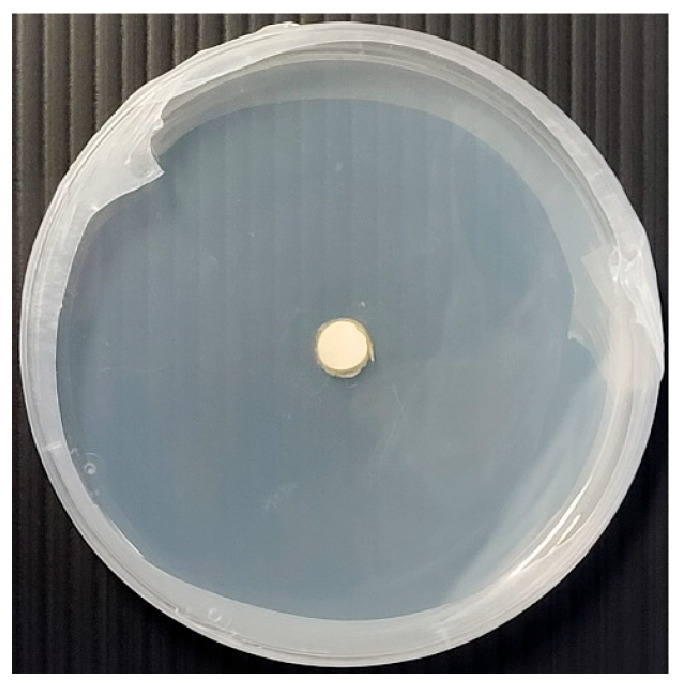
Freshly plated circular 8 mm diameter hyphal plug used for experimentation.

For each experimental series, mycelial growth and morphology relating to all genotypes of interest (APE, B+, JMF, and PA) were tracked. In all cases, circular 8 mm diameter hyphal plugs were taken from parental cultures and plated on fresh media (Figure 1) corresponding to the appropriate treatment for each experimental series. Each experimental series included four replicates per genotype of interest. Plates were wrapped in parafilm and left in the dark at room temperature to colonize plates for 25 days. Cultures were examined every 5 days and photos were taken using a Samsung Galaxy S21 FE 5G (Samsung Electronics Canada, Missisauga, ON, Canada) for further analysis.

### 2.3. Growth Assessment

Mycelial growth was tracked every 5 days for 25 days. Mean colony surface area (mm^2^) was extracted from previously obtained images using ImageJ software version 1.54 [19]. To track mycelial growth trends for each experimental series, surface areas of each genotype grown on each media were graphed using Microsoft Excel (Redmond, WA, USA). Significances in periodic surface area growth were conducted using ANOVA with Tukey HSD post hoc analysis using R Statistical Software version 4.5.0 (Vienna, Austria). In all cases, four replicates were considered per treatment when possible. However, for Experimental Series 2, due to the occurrence of bacterial contamination, some replicates were excluded for a minimum of three replicates per treatment and are described as follows. For B+ on PDA, three replicates were included from days 5–25. For JMF on PDA, three replicates were included from days 10–25. For PA on PDA R, three replicates were included from days 20–25.

### 2.4. Morphology Assessment

**Figure 2:**
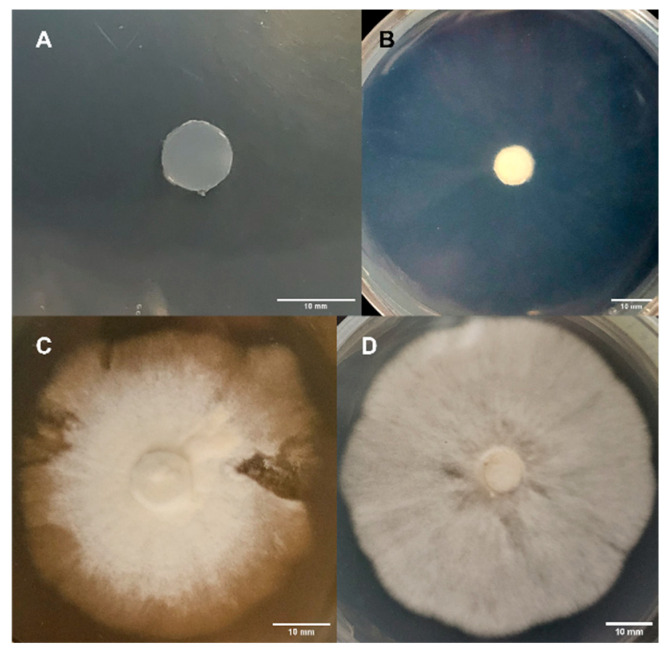
Rankings of mycelial morphology after 25 days: (**A**) no growth [rank 0], (**B**) sparse mycelium [rank 1], (**C**) sparse mycelium becoming dense [rank 2], and (**D**) dense mycelium [rank 3].

Mycelial morphologies were ranked on day 25. Rankings were assigned based on density of mycelium colonies, including 0 (no growth), 1 (sparse mycelium), 2 (sparse mycelium becoming dense), and 3 (dense mycelium) (Figure 2). To determine if there were correlations between media and morphology, Pearson correlation was used to calculate correlation coefficient. Analyses were conducted using Microsoft Excel (Redmond, WA, USA).

**Figure 3:**
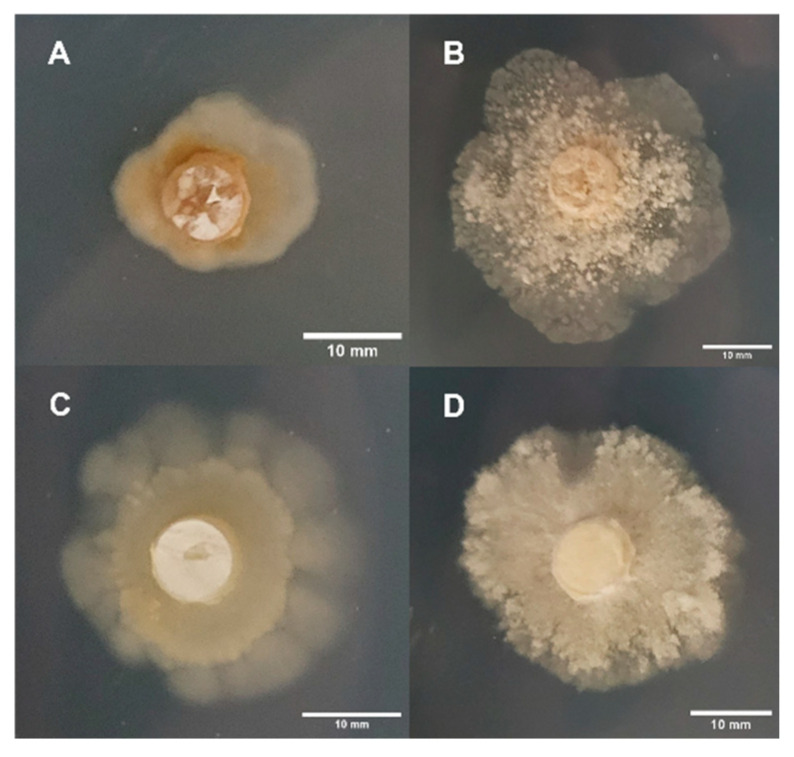
Irregular mycelial morphologies on MS after 25 days: (**A**) APE, (**B**) B+, (**C**) JMF, and (**D**) PA.

In all instances, due to the irregular morphology associated with mycelium growth on MS, this treatment was excluded from the morphology assessments for Experimental Series 1 (Figure 3). Four replicates were considered per treatment when possible. However, due to the occurrence of bacterial contamination in Experimental Series 2, some replicates were excluded for a minimum of three replicates per treatment. Reductions in replicates were the same as those described for the growth rate analysis.

## 3. Results

### 3.1. Experimental Series 1

Experimental Series 1 focused on all genotypes of interest (APE, B+, JMF, and PA). Each genotype of interest was cultured on PDA, MEA, MS, and WA. Growth was tracked for 25 days and morphologies were assessed on day 25.

#### 3.1.1. Growth Trends on Day 25

Colonization trends on day 25 are shown in Table 2. No significant differences were observed for APE on WA vs. MEA. These two were significantly higher than PDA, which was significantly higher than MS. Results corresponded to averages of 100%, 30.5%, 95.8%, and 4.0% colonization of the Petri plate surface areas for the WA, PDA, MEA, and MS treatments, respectively (Table 2). For B+, the WA, PDA, and MEA growth rate differences were insignificant with 100% of the plate surfaces occupied by day 25. However, WA, PDA, and MEA were all significantly greater than MS, which averaged 15.7% plate coverage (Table 2). For JMF, all media treatments were significantly different by day 25. The highest mean surface area was with WA, followed by MEA, PDA, and MS, corresponding to 95.6%, 64.2%, 55.4%, and 9.2% colonization, respectively (Table 2). After 25 days, the growth rate of PA on WA showed no difference compared to PDA, though both were significantly higher than both MEA and MS, and MEA was significantly higher than MS. For the WA, PDA, MEA, and MS treatments, average percent plate coverage was 61.8%, 69.7%, 47.8%, and 14.2%, respectively (Table 2).

#### 3.1.2. Growth Response 5-Day Trends of APE

**Figure 4:**
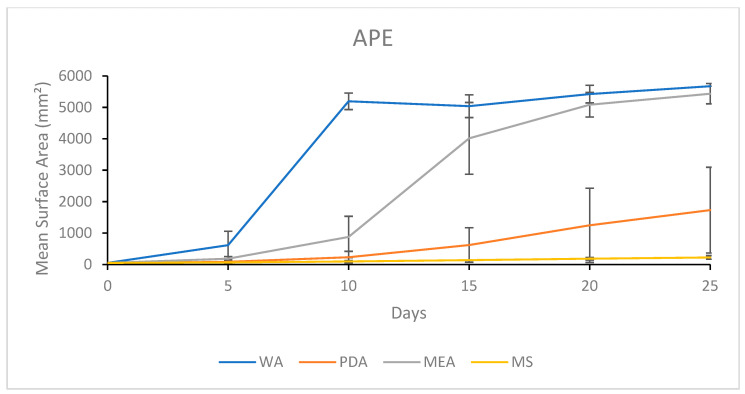
Surface area colonization 5-day trends of APE. Error bars represent standard deviation.

The mean surface area of APE grown on WA was significantly higher than all other treatments by day 5. By day 10, WA remained significantly higher than all other treatments, MEA was significantly higher than MS, while there was no significant difference between MEA and PDA. By day 15, there were no significant differences between MEA and WA, though these treatments were significantly higher than PDA and MS, which were insignificant. This trend continued until day 25. The surface area growth trends of APE on different media treatments are shown in Figure 4. By day 25, PDA allowed significantly higher surface area growth than MS, which were both significantly lower than both WA and MEA, showing no significant differences (Figure 4). The results of the Tukey HSD post hoc analysis are available in Table S1.

**Figure 5:**
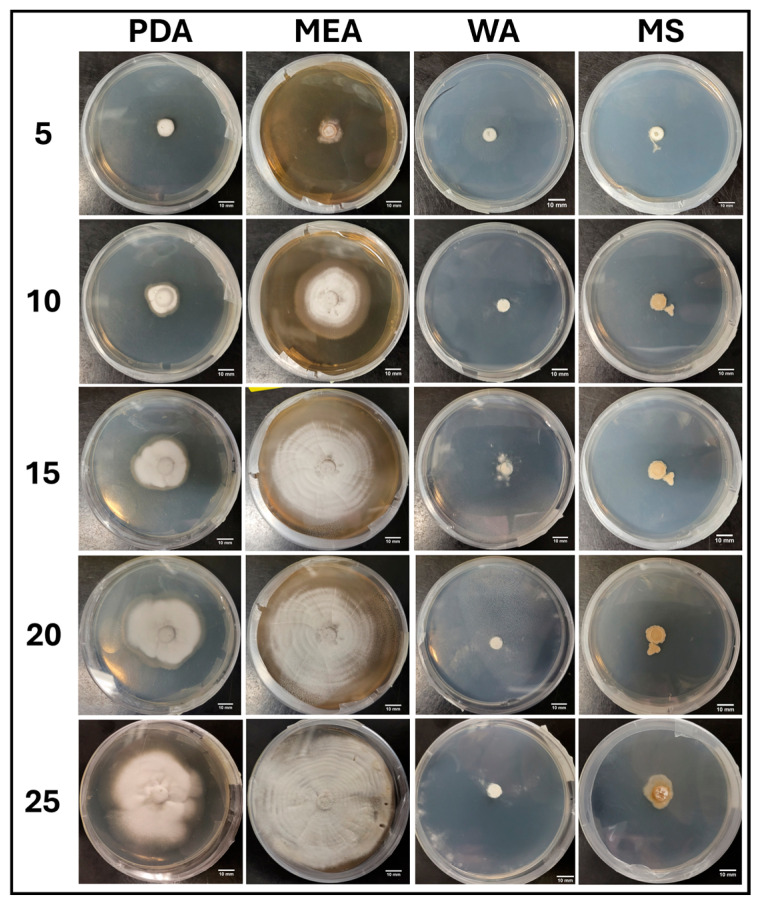
Representations of APE colony formation on different media throughout the 25-day experimental series.

Visual representations of APE colony formation on media treatments are presented in Figure 5. Slow growth and irregular morphology were seen on MS, while WA afforded a fast colonization rate, but sparse morphology. Alternatively, PDA allowed healthy looking mycelia, but the colonization rate was slower compared to that of MEA. MEA allowed rapid colonization and healthy morphology (Figure 5).

#### 3.1.3. Growth Response 5-Day Trends of B+

**Figure 6:**
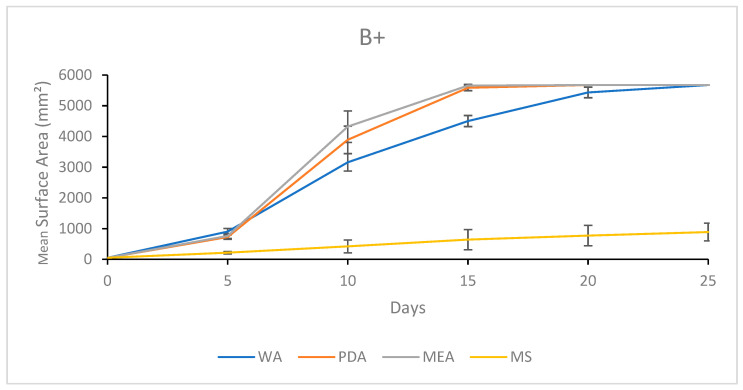
Surface area colonization 5-day trends of B+. Error bars represent standard deviation.

After 5 days of growth, the mean surface areas of B+ on MEA, PDA, and WA were significantly greater than that of MS. This trend continued to day 25. By day 10, there were no significant differences between WA and PDA or PDA and MEA, though MEA was significantly greater than WA. Day 15 showed no significant difference between PDA and MEA, though WA was significantly lower than both. Thereafter, no significant differences were observed between the MEA, PDA, or WA treatments for the remainder of the experiment (Figure 6). The results of the Tukey HSD post hoc analysis are available in Table S2.

**Figure 7:**
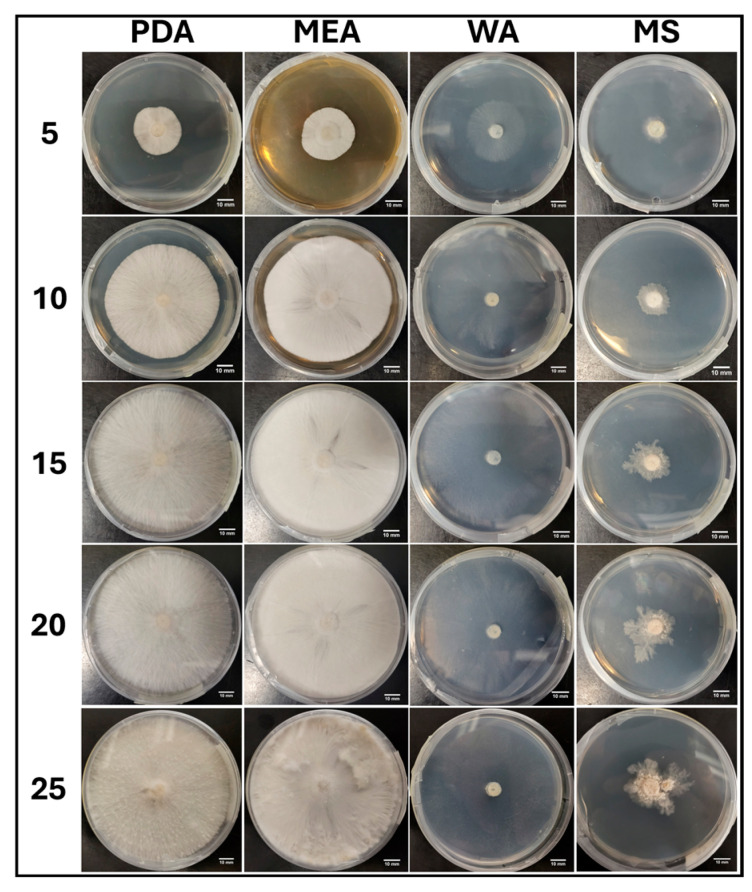
Representations of B+ colony formation on different media throughout the 25-day experimental series.

Visual representations of B+ colony formation on different media are presented in Figure 7. While both PDA and MEA allowed colonization at similar rates, MEA showed more a tomentose morphology compared to the more reticulate morphology observed in the PDA treatment. In general, both PDA and MEA allowed full colonization by day 20, while it generally took the full 25 days for the WA treatments to fully colonize the plates. Sparse mycelium was a characteristic of WA. Mycelia grown on MS showed slow development and appeared translucent and off-white, suggesting irregular development (Figure 7).

#### 3.1.4. Growth Response 5-Day Trends of JMF

**Figure 8:**
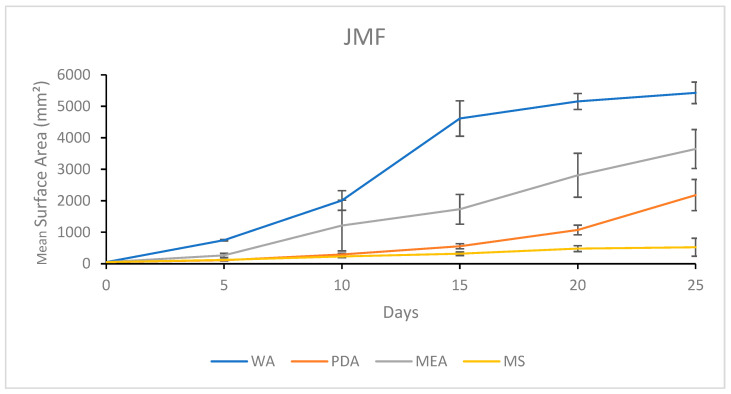
Surface area colonization 5-day trends of JMF. Error bars represent standard deviation.

The growth of JMF showed no significant differences between PDA and MS treatments after 5 days, although MEA was significantly greater than both, while WA was significantly greater than all other treatments. By day 10, PDA and MS continued to show no significant differences, while WA and MEA also showed no significant differences, but they both allowed significantly greater growth than MS and PDA. After 15 days, WA was significantly greater than MEA, which were both significantly greater than PDA and MS, showing no significance. This trend continued until day 25 when all treatments were significantly different, showing WA > MEA > PDA > MS (Figure 8). The results of the Tukey HSD post hoc analysis are available in Table S3.

**Figure 9:**
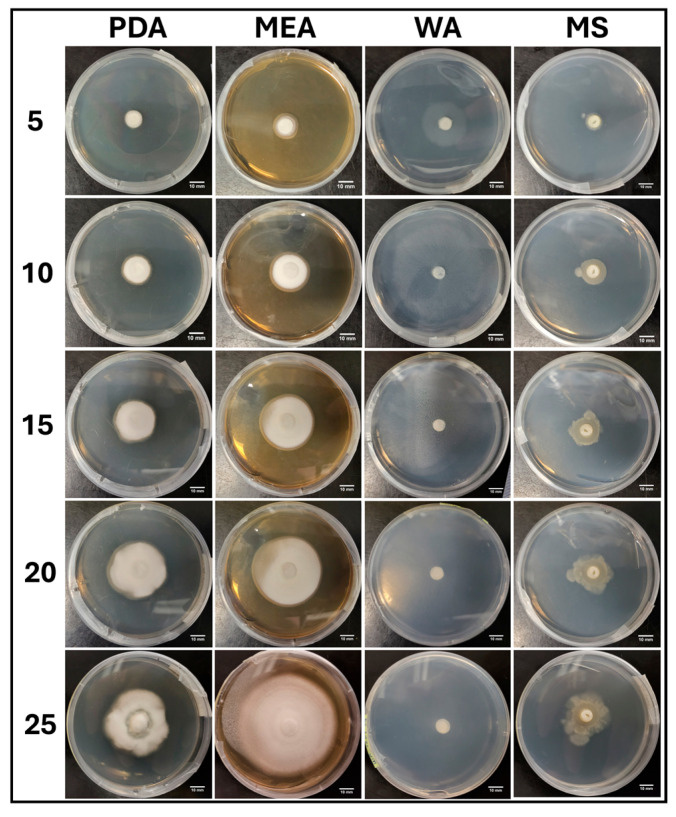
Representations of JMF colony formation on different media throughout the 25-day experimental series.

The colony formation of JMF is presented in Figure 9. The fastest colonization and a sparse morphology were characteristics of WA, while MEA allowed faster colonization compared to PDA with a more uniform distribution of mycelium growth. Slow growth was characteristic of MS with mycelia developing an atypical yellow translucent morphology (Figure 9).

#### 3.1.5. Growth Response 5-Day Trends of PA

**Figure 10:**
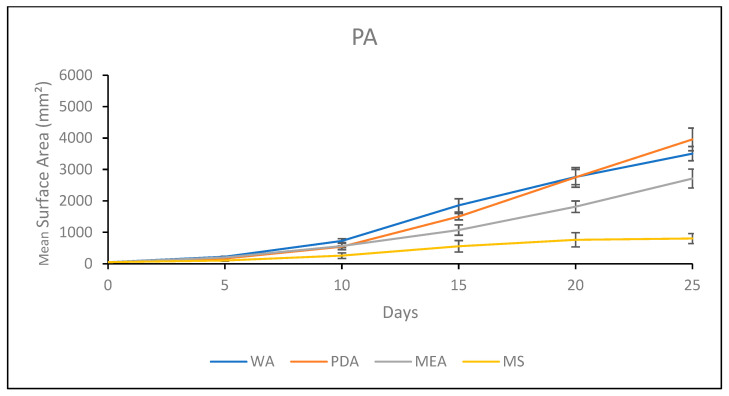
Surface area colonization 5-day trends of PA. Error bars represent standard deviation.

By day 5, MEA was not significantly different than WA or PDA. However, WA and PDA were significantly different, and MS showed significantly lower colonization than all the other treatments. This trend continued until day 15, where all treatments were significantly different, with WA > PDA > MEA > MS. Day 20 showed no significant differences between PDA and WA, while MEA was significantly lower than PDA and WA, and MS was significantly lower than MEA. This trend continued for the rest of the experiment (Figure 10). The results of the Tukey HSD post hoc analysis are available in Table S4.

**Figure 11:**
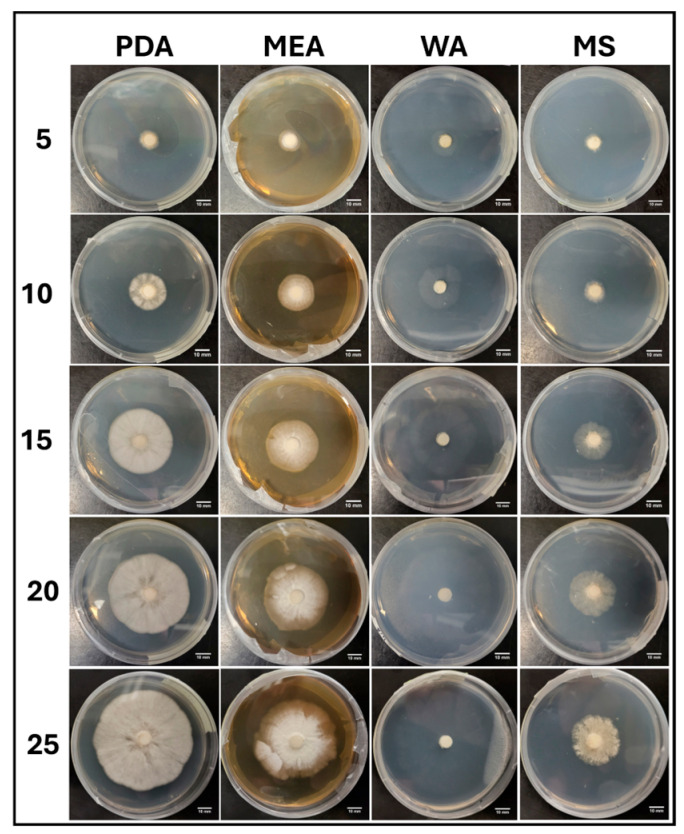
Representations of PA colony formation on different media throughout the 25-day experimental series.

Visual representations of PA colony formation are presented in Figure 11. Fast colonization of the healthy, thick mycelium with a slight tomentose morphology was a characteristic of PA on PDA. Mycelial development on MEA began sparse and became thick and tomentose as development continued. The fastest rate of colonization occurred on WA, which grew sparse mycelium. Growth on MS was slow, with sparse mycelium that eventually developed thick patches (Figure 11).

#### 3.1.6. Morphology Assessments

As described in Figure 2, morphologies at day 25 for Experimental Series 1 are presented in Table 3. Mycelia produced by WA showed 100% sparse morphology (rank 1). This result held true for all isolates grown on WA. When APE was grown on PDA, 75% showed the sparse-becoming-dense morphology (rank 2), while 25% produced dense mycelia (rank 3). For APE on MEA, 50% showed the sparse-becoming-dense morphology (rank 2), while 50% showed the dense morphology (rank 3). Dense mycelium (rank 3) was characteristic for 75% of B+ on PDA, while 25% showed sparse mycelium with the becoming-dense morphology (rank 2). On MEA, 75% of B+ developed dense mycelium (rank 3) while 25% showed sparse mycelium with the becoming-dense morphology (rank 2). The dense mycelium morphology (tank 3) was characteristic of 100% of JMF and PA mycelia grown on PDA. On MEA, 100% of PA showed the sparse-becoming-dense morphology (rank 2), while 100% of JMF mycelia showed the dense morphology (rank 3) (Table 3).

No clear relationships were found between PDA or MEA and morphology distribution (Table 4). The results showed media treatments to be insignificant indicators of variability among mycelial morphologies.

### 3.2. Experimental Series 2

Experimental Series 2 focused on all the genotypes of interest (APE, B+, JMF, and PA). Each was cultured on PDA, PDA P, PDA R, and WA. Growth was tracked for 25 days, and emergent morphologies were assessed on day 25.

#### 3.2.1. Growth Trends on Day 25

Colonization trends on day 25 are shown in Table 5. No significant differences were observed for APE grown on PDA P, PDA, or WA. However, the mycelium failed to colonize the PDA R plates. Treatments of PDA P, PDA, WA, and PDA R resulted in 83.7%, 72.3%, 95.0%, and 0% colonization, respectively (Table 5). The mycelial growth of B+ showed no significant differences on day 25, with PDA P, PDA, and PDA R resulting in 100% colonization, and WA showing 97.7% colonization (Table 5). After 25 days of growth, there were no significant differences between JMF on PDA P and PDA, although both were significantly greater than PDA R. On WA, mycelial growth was significantly greater than that of all other treatments. The average colonization of Petri plate surfaces was 59.5%, 49.1%, 97.5%, and 32.0% for PDA P, PDA, WA, and PDA R, respectively (Table 5). For PA, PDA P and PDA showed no significant differences on day 25. Both PDA P and PDA were significantly greater than WA and PDA R, which showed no significant differences. The average percentages of Petri plate colonization based on treatment included 96.4%, 95.2%, 81.3%, and 82.5% for PDA P, PDA, WA, and PDA R, respectively, (Table 5).

#### 3.2.2. Growth Response 5-Day Trends of APE

**Figure 12:**
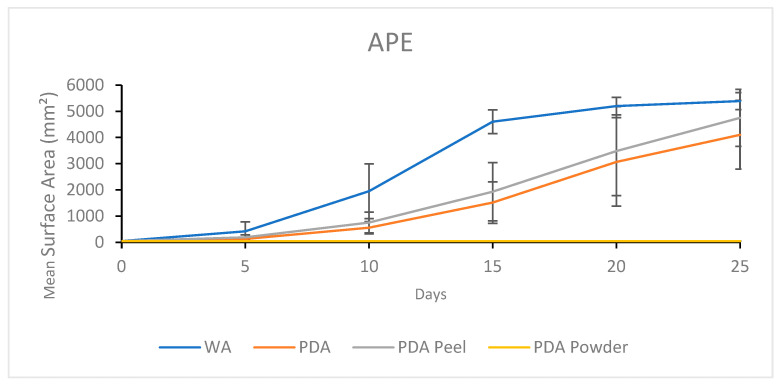
Surface area colonization 5-day trends of APE. Error bars represent standard deviation.

No significant differences between treatments occurred for APE until the 10th day of growth which showed that WA was significantly greater compared to PDA and PDA R. By day 15, WA was significantly greater than all other treatments, while PDA P and PDA R showed no significant differences. On day 20, there were no significant differences between WA, PDA P, and PDA, although they all allowed significantly greater colonization than PDA R. This trend continued until the end of the experiment (Figure 12). The results of the Tukey HSD post hoc analysis are presented in Table S5.

**Figure 13:**
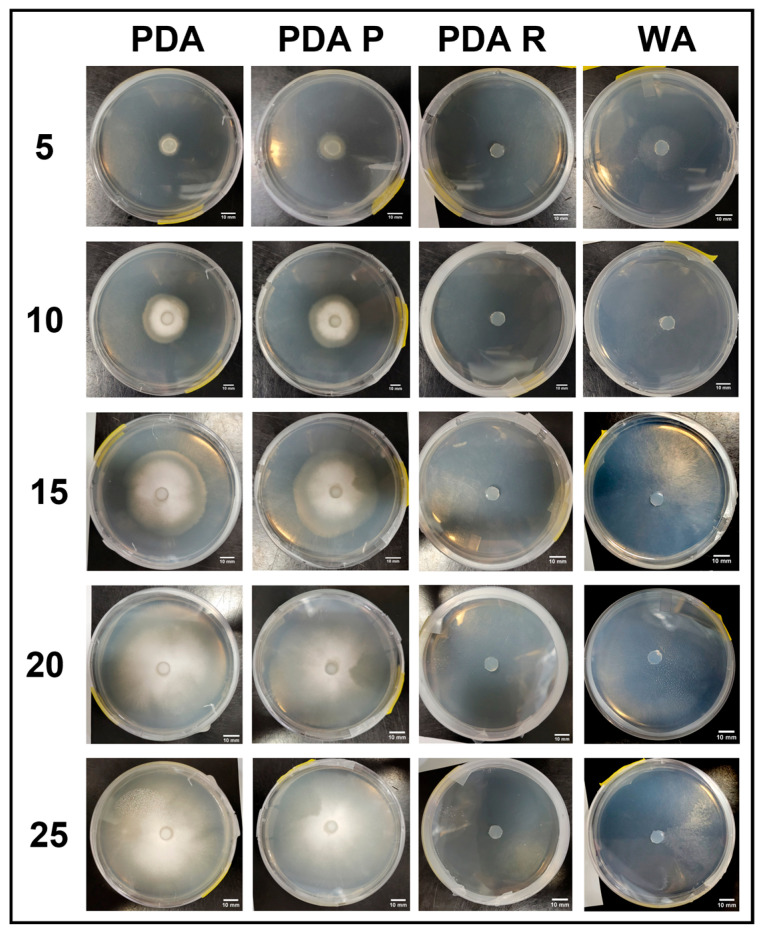
Representations of APE colony formation on different media throughout the 25-day experimental series.

Visual representations of APE colony formation are shown in Figure 13. Similar morphologies and fast colonization were characteristic of PDA and PDA P. Mycelia did not proliferate on PDA R, while WA allowed rapid colonization and a sparse morphology (Figure 13).

#### 3.2.3. Growth Response 5-Day Trends of B+

**Figure 14:**
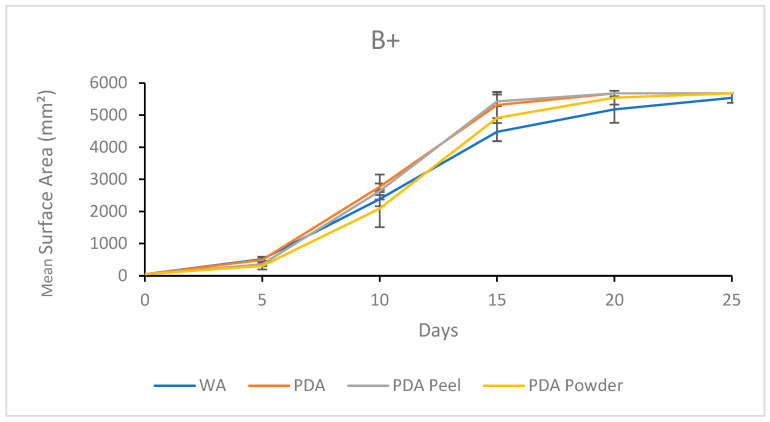
Surface area colonization 5-day trends of B+. Error bars represent standard deviation.

By day 5, the mean surface area on WA was greater than that on PDA P and PDA R. No significant differences between WA and PDA or between PDA P, PDA, and PDA R occurred. Day 10 showed no significant differences between treatments. This trend continued for the rest of the experiment (Figure 14). The results of the Tukey HSD post hoc analysis are presented in Table S6.

**Figure 15:**
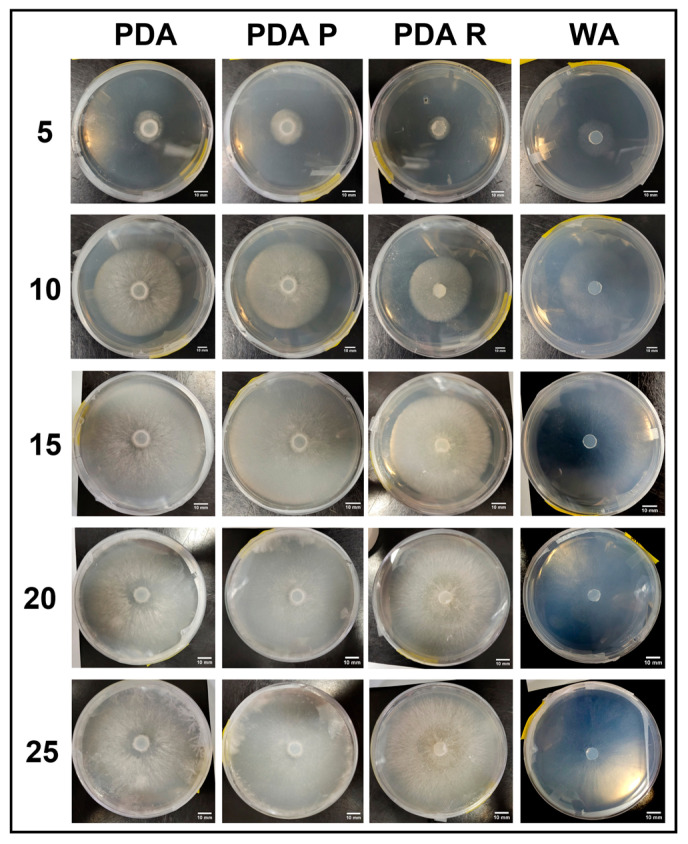
Representations of B+ colony formation on different media throughout the 25-day experimental series.

Visual representations of B+ are shown in Figure 15. Quick colonization occurred on PDA and PDA P compared to on PDA R and WA. Mycelial morphology was reticulate on PDA and PDA R compared to the tomentose morphology on the PDA P plates. Mycelia grown on PDA R developed a yellow tinge, while WA allowed sparse mycelial growth. Full colonization occurred on PDA and PDA P no later than day 20 of growth. The mycelia of these treatments began extending up the sides of the plates and growing on the underside of the plate lids, which was not seen with the WA or PDA R treatments (Figure 15).

#### 3.2.4. Growth Response 5-Day Trends of JMF

**Figure 16:**
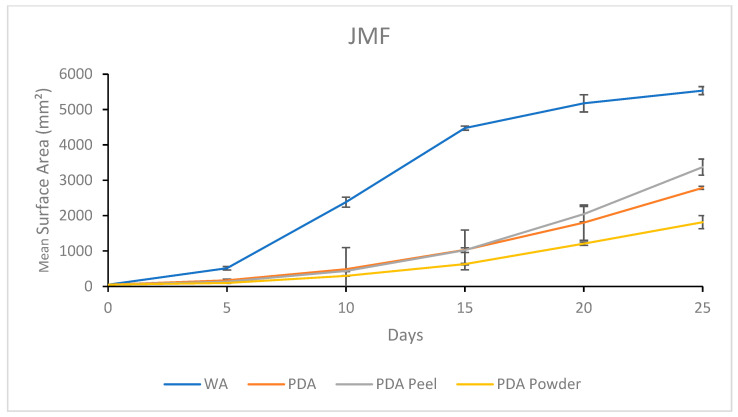
Surface area colonization 5-day trends of JMF. Error bars represent standard deviation.

The growth of JMF on WA was significantly greater than PDA, PDA P, or PDA R by day 5, while PDA, PDA P, and PDA R showed no significant differences. This trend continued until day 15 when PDA and PDA P became significantly greater than PDA R. By day 20, the growth rates between PDA, PDA P, and PDA R were insignificant, while WA was significantly greater than all PDA treatments. By day 25, WA was significantly greater than all other treatments, while PDA P and PDA were insignificant, but significantly greater than PDA R (Figure 16). The results of the Tukey HSD post hoc analysis are available in Table S7.

**Figure 17:**
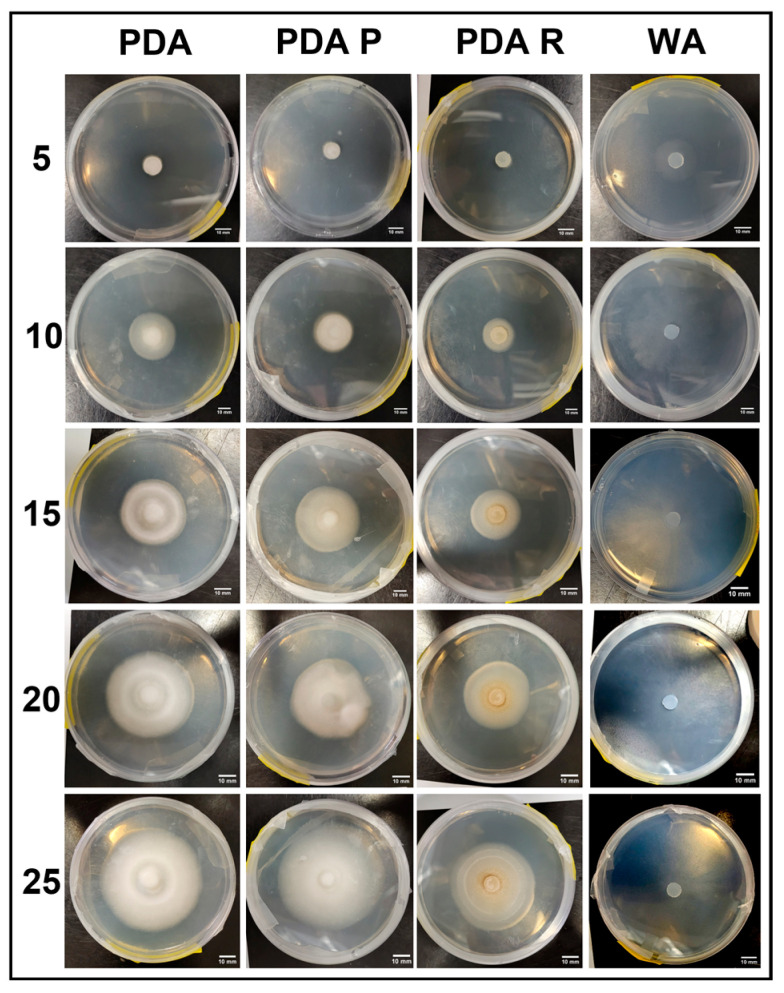
Representations of JMF colony formation on different media throughout the 25-day experimental series.

Visual representations of JMF are shown in Figure 17. Thick, white mycelium was associated with both PDA and PDA R, which allowed uniform growth from the central inoculation point. Mycelium grown on the PDA R was associated with a yellow tinge that developed at the central inoculation point and eventually extended toward the colony tips. Sparse mycelia and fast growth were characteristic on WA (Figure 17).

#### 3.2.5. Growth Response 5-Day Trends of PA

**Figure 18:**
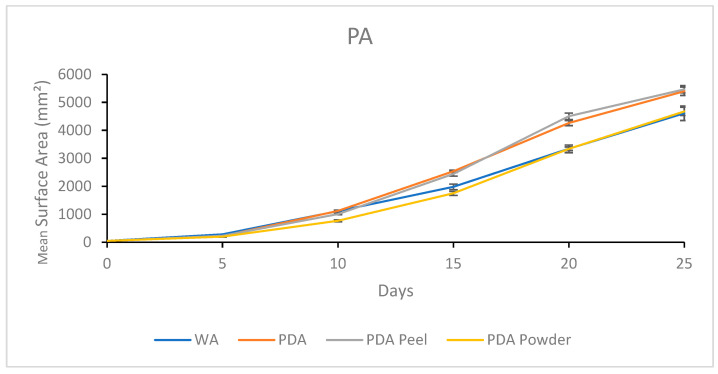
Surface area colonization 5-day trends of PA. Error bars represent standard deviation.

Day 5 of growth showed no significant differences between PA mycelium treatments. By day 10, PDA R was significantly lower than all other treatments, and there were no significant differences between PDA, PDA P, or WA. Day 15 showed no significant differences between PDA and PDA P, although these treatments were significantly greater than WA and PDA R, with no significant differences among themselves. This trend continued until day 25 when all PDA treatments showed insignificant differences, and WA was significantly different than PDA P and PDA (Figure 18). The results of the Tukey HSD post hoc analysis are available in Table S8.

**Figure 19:**
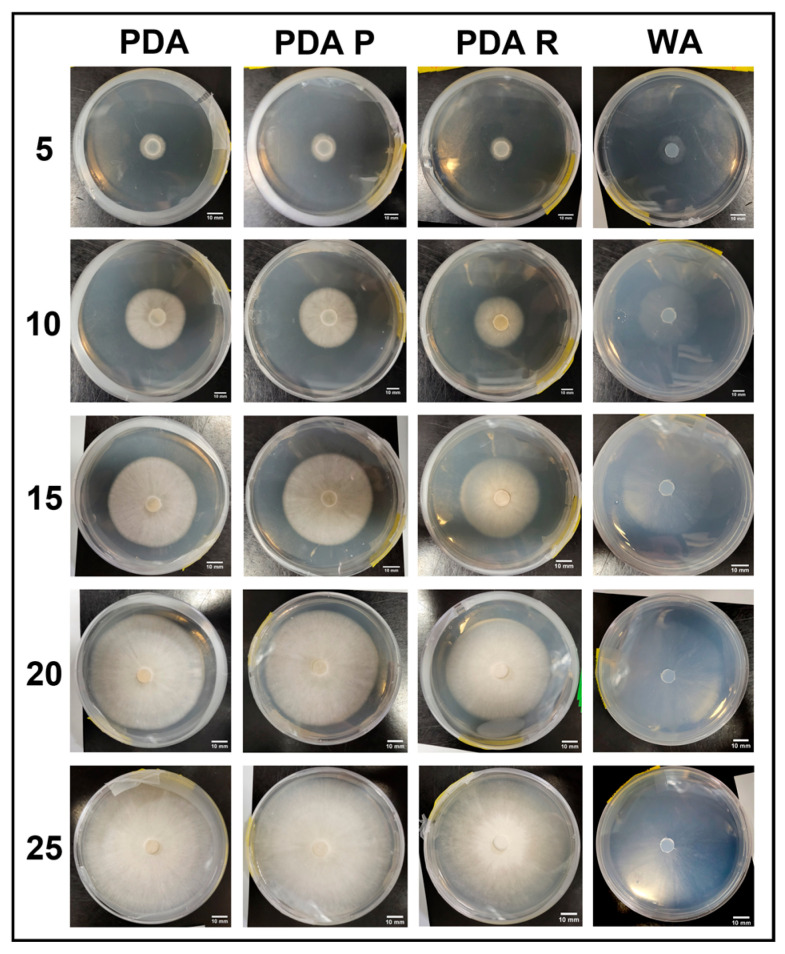
Representations of PA colony formation on different media throughout the 25-day experimental series.

Visual representations of PA are shown in Figure 19. Overall, PDA and PDA R appeared to allow similar growth and morphology characteristics. Slower growth was observed on WA and PDA R compared to on PDA and PDA P. Sparse mycelium was characteristic of WA allowance, and white PDA R mycelium appeared dense with a yellow tinge compared to mycelia grown on PDA and PDA P (Figure 19).

#### 3.2.6. Morphology Assessments

As described in Figure 2, the morphologies on day 25 of growth associated with Experimental Series 2 are presented in Table 6. A sparse morphology (rank 1) was characteristic of all cultures of all genotypes grown on WA. After 25 days, 100% of APE mycelium showed sparse mycelium becoming dense (rank 2) on both PDA P and on PDA. However, 100% APE cultures failed to grow on PDA R (rank 0). Dense mycelium (rank 3) was characteristic of 100% of B+ grown on PDA P, PDA, and PDA R. Sparse-becoming-dense mycelia (rank 2) were characteristic of 75% of JMF on PDA P, while 25% had dense mycelium (rank 3). On PDA, 67% of JMF developed sparse-becoming-dense mycelium (rank 2), while 33% showed dense mycelium (rank 3). On PDA R, 50% of JMF displayed sparse-becoming-dense mycelium (rank 2), while the other 50% developed dense mycelium (rank 3). Dense mycelia (rank 3) were characteristic of 100% of PA on both PDA P and PDA, while on PDA R, 67% showed a dense morphology (rank 3) and 33% developed a sparse-becoming-dense morphology (rank 2) (Table 6).

A moderate positive correlation was found between PDA P and mycelial density based on the correlation coefficient of 0.53, and although the coefficient of determination was low (0.28), the *p*-value of 0.03 was significant. Similarly, the PDA correlation coefficient of 0.41 indicated a moderate positive correlation with morphology. However, the low coefficient of determination (0.17) and high *p*-value (0.15) did not support a relationship between PDA and mycelial density. With a correlation coefficient of 0.82, a strong positive correlation was found between PDA R and the morphology rating. Additionally, a coefficient of determination of 0.69 and *p*-value of 0.0001 identified PDA R to be correlated with morphology (Table 7).

### 3.3. Observations Related to Contamination

**Figure 20:**
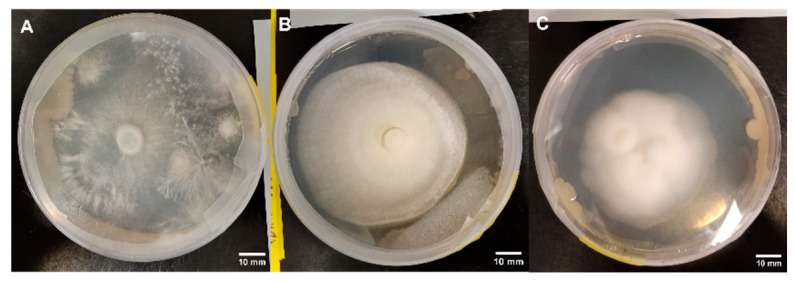
Response of specific genotypes to contamination. (**A**) B+ grown on PDA, (**B**) PA grown on PDA R, and (**C**) JMF grown on PDA are shown.

While no contamination was observed in Experimental Series 1, three replicates developed bacterial contamination during Experimental Series 2, including B+ on PDA (Figure 20A), PA on PDA R (Figure 20B), and JMF on PDA (Figure 20C). The genotypes of interest responded differently to the bacterial contaminant. In response to contamination, B+ developed rhizomorphic mycelium that grew over the bacterial colonies (Figure 20A), PA focused growth toward the opposite direction of the bacteria (Figure 20B), and JMF developed clumps of dense, tomentose mycelium that began to grow upward and opposite the contaminant (Figure 20C).

## 4. Discussion

These genotypes used in the current work were chosen based on specific mycelial growth characteristics described in the grey literature. Particularly, APE is considered to be among the slowest growing *P. cubensis* genotypes. Conversely, B+ mycelium is described as among the fastest growing of the commonly cultured *P. cubensis* genotypes. Additionally, the speed of JMF mycelium growth is thought to be intermediary among the commonly cultured *P. cubensis* genotypes. While the former three are generally considered coprophilous genotypes [20], PA is a wood-rotting species [21]. Similarly, the selected media were chosen because they represent commonly used or commonly available media for the growth of Basidiomycetes.

Developing effective production practices for filamentous fungi centers around selecting an effective basal medium that supports the growth, yield, and target metabolite production, then optimizing nutritional profiles to better suit specific genotypes [8]. The current investigation showed that MEA has a particular advantage in supporting the growth of APE and JMF mycelium, followed by PDA treatments prepared from raw ingredients (PDA and PDA P). Similar to these results, the mycelium growth rate and density of *Pleurotus sapidus* is greatest when grown on malt extract- and potato dextrose-based media [11]. Additionally, malt extract, along with alternative grain- and potato-based media such as sweet potato extract and oat extract media, can improve the mycelium growth of *Lentinula edodes* over PDA [22]. Malt-based extracts used for mycology are high in saccharides such as maltose [23] in addition to being rich in vitamins, amino acids, and coenzymes that help support fastidious growth [14]. Interestingly, in both experimental series, the mean growth of PA mycelia was significantly greater on PDA treatments made from raw ingredients from the 15th day of growth until the 25th day compared to on all other treatments. This could be due to the wood-rotting nature of PA [21] which differs from the coprophilous nature of the other genotypes of interest [20]. As a testament to the vigorous growth associated with B+, in all cases, MEA, PDA, and PDA P allowed for the full colonization of the Petri plates by the 20th day of growth and the full colonization of PDA R plates by the 25th day. However, the concentration of malt extract in the media can also greatly impact the mycelium growth and density of different genotypes of filamentous fungi [24], but this was not examined in the current study. It is possible that an optimized concentration of malt extract may have further enhanced the growth of B+ and improved the growth of PA to a comparable level as the PDA treatments made from raw ingredients. This is an important aspect to explore when conducting future media optimization studies.

Media selection for the proliferation of quality mycelium is an important consideration for different types of fungi, including *Psilocybe* spp. Although faster growing genotypes like B+ largely showed no differences related to growth between media treatments, slower growing genotypes such as APE showed substantial differences in growth rates between media treatments. Thus, cultivation systems should be carefully evaluated to suit particular genotypes. While the fastest growth rates of the *P. cubensis* genotypes occurred on MEA, PDA treatments prepared from raw ingredients were associated with rapid PA colonization. This discrepancy is likely due to the distinct nutritional requirements of coprophilous vs. wood-rotting mushrooms, which have evolved to grow on different substrates. Media carbon and nitrogen sources, CN ratios, and vitamin contents are significant factors impacting fungal growth [8]. In the wild, PA grows on woody debris [21] which has a high CN ratio [25], while *P. cubensis* has been found on cow dung [20], containing a lower CN ratio compared to that of wood [26]. Similarly, the CN ratio of MEA is low compared to that of PDA [8]. Nitrogen availability can negatively impact the growth rates of certain hard-wood mushroom species, although the occurrence of micronutrients, vitamins, and simple carbohydrates does not necessarily reflect the growth rates to the same degree [27]. Wood debris is largely made up of cellulose, hemicellulose, and lignin [28], which are converted into sugars for fungal growth. While PDA contains carbohydrates, minerals, and vitamins [29], it is still considered a basic or narrow-range media [9,12] compared to other commonly used formulations. Alternatively, cow dung is a rich source of crude protein, fiber, lipids, and macro- and micronutrients [30]. Although the relative amounts or carbohydrates in MEA are higher than in cow dung and the overall concentrations of proteins and mineral nutrients in cow dung are higher than in MEA, the total available nutrients in these substrates are similar [30,31]. While lower concentrations of mineral elements are essential for the growth of certain fungi, higher concentrations can be toxic [8]. Thus, it is likely that PA mycelium requires more carbohydrates and less additional nutrients to promote growth and healthy mycelium development compared to *P. cubensis* genotypes, which likely require more vitamins, amino acids, and mineral nutrients. These insights can be used to tailor media treatments in the creation of optimized media formulations for the different genotypes of interest.

Although in most cases the growth rates of mycelium between PDA P and PDA treatments were similar, the general trend for surface area colonization was favoured by PDA P. Potato peels contain additional antioxidants, vitamins, proteins, lipids, and carbohydrates that may not be held within the potato flesh [32]. This might positively influence growth rates. Additionally, sulfur and chloride are mineral elements contained in the potato flesh in equal or higher concentrations than found in the peels [33]. Sulfuric [34,35] and chloridic [36,37] compounds can inhibit mycelium growth in high concentrations, although it is not likely that the concentrations found in potato flesh would be detrimental to mycelium growth. Peels also contain higher concentrations of calcium, zinc, and iron than the potato flesh [33]. Calcium sources can promote hyphal branching [38], while iron can promote the accumulation of additional mineral elements that support fungal growth [39]. Almjalawi et al. [15] found that media prepared using 5 and 10 g/L dried potato peels enhanced the mycelium growth rates of various gourmet mushroom genotypes compared to commercial PDA. The aforementioned results are in line with the results of the current experiment which shows that PDA P allowed significantly higher growth rates than PDA R. This finding can likely be attributed to higher concentrations of media components, such as vitamins, which may be present in the potato peels and could contribute to the optimization of customized media formulations. Future studies should aim to confirm this supposition.

Experimental Series 2 demonstrated that mycelium grown on PDA treatments from raw ingredients outperformed mycelium grown on commercial PDA R. Similarly, Kalaw et al. [40] found that media formulations made from raw natural ingredients favoured the mycelium growth of various macrofungi compared to commercial PDA powder. Different commercial manufacturing processes for PDA could contribute to variability in nutritional compositions [13], resulting in batch variability and inconsistencies between suppliers [9]. Although batch variations are also conceivable with media made using raw ingredients, the current results show that preparing PDA from raw ingredients supports the mycelium growth of *Psilocybe* spp., perhaps to an even greater degree when the peel is kept intact. To validate this supposition, an experiment should be conducted that compares PDA, PDA P, and PDA R at a gradient of different concentrations.

With the more socially acceptable view of psychoactive natural products such as psilocybin-producing mushrooms [1] and cannabis [41], accessibility has increased due to changes in legislation and commercialization. However, mushrooms produced for consumption should adhere to GACP and GMP guidelines. This can be challenging, considering potential inconsistencies associated with media such as MEA and PDA. Among the most commonly used media formulation for plant tissue culture is MS [42], which is carefully formulated to include only specific ingredients (mineral nutrients, vitamins, amino acids, etc.) at precise concentrations. This more synthetically derived type of media ultimately reduces batch variability to potentially produce more consistent mycelia. Thus, MS was included as a treatment for Experimental Series 1. However, mycelium growth on MS resulted in irregular morphologies and significantly lower growth rates than all other treatments for each genotype of interest. The mycelium growth rates of various gourmet mushrooms were also found to be lower on MS compared to on other treatments, including PDA [43]. Alternatively, Nasim et al. [44] found that MS allowed higher oyster mushroom mycelium colonization rates than PDA, although MEA outperformed both MS and PDA treatments. One major difference between that study and the current work is the addition of vitamins to the MS media, which was not included in Experimental Series 1. Certain vitamins can influence growth responses, acting as coenzymes to catalyze various cellular functions [18], the lack of which might have contributed to the low mycelium growth rates observed in Experimental Series 1. Alternatively, the relatively high concentrations of potentially toxic elements such as chloride present in MS could have contributed to the delayed growth rate and irregular morphologies seen in Experimental Series 1. Hence, it is recommended that individuals interested in *Psilocybe* spp. production opt for MEA or PDA produced from raw ingredients as a means to proliferate mycelium, rather than using basal media formulations designed for plant tissue culture. Additionally, future experiments aiming to optimize GMP- and GACP-compliant media formulations should consider the concentrations of mineral nutrients, vitamins, and amino acids as important media additives.

Environmental stimuli, such as nutrient availability, can influence variability in mycelium morphology, leading to the induction of specific growth strategies [45]. Basidiomycete mycelium begins growing isotropically until interconnected networks branch off toward a path of nutrient abundance [24]. The morphologies of these networks largely depend on the emergent growth strategy. Two contrasting mycelium growth strategies have been described as *phalanx*, whereby dense hyphal mats steadily grow in response to easily accessible nutrients, and *guerilla*, where exploratory or opportunistic hyphae stretch in search of nutrients [24,45]. Examples of these different growth strategies can be seen in the current work. For instance, mycelium grown on WA developed fast-growing, sparsely branched mycelia, which is in contrast to the dense mycelia generally produced on MEA treatments. More cottony mycelium morphologies are characteristic of growth on nutrient-rich media for many fungal species [14] Although the weak positive correlation between PDA or MEA and morphology were insignificant for Experimental Series 1, these treatments still allowed healthy, dense mycelia for each genotype of interest. Similarly, the moderate correlation between morphology occurrence and PDA was not statistically significant. However, there was a significant moderate correlation between morphology and PDA P which was associated with healthy, dense mycelia and a significant strong positive correlation between morphology and PDA R. Although PDA R did support the growth of B+, JMF, and PA, the mycelia were discoloured, and PDA R did not support the growth of APE. Westphal et al. [13] found that commercial PDA influenced the secondary metabolite production of *Fusarium* spp., resulting in variations in mycelium colour, although there was no solitary nutrient-related regulatory mechanism found to explain these findings. The failure of PDA R to support APE growth may have been due to the origin cultures of Experimental Series 2, which were grown on WA. Since APE is a slower growing genotype, the mycelia might not have had enough nutrients sequestered to survive the transfer to PDA R. It is thus suggested that *Psilocybe* spp. growers put careful consideration into which commercial media formulation they use for mycelium proliferation, especially in the case of slow-growing genotypes like APE.

It is interesting to observe the differential responses of the genotypes to bacterial contamination. Bacterial interactions can influence the developmental features of mycelia, including growth dynamics and morphology [46]. Certain documented interactions include hyphae-mediated bacteriolysis, chemotaxis-mediated mycelia–bacteria connections, the enzymatic promotion or inhibition of hyphal growth, or the modification of the mycelium architecture to promote strand development or fruiting body initiation [47]. However, there is limited understanding of the underlying mechanisms of these interactions as they pertain to Basidiomycetes [46]. It is only possible to speculate about the nature of mycelial responses to contamination since the bacteria in Experimental Series 2 were not identified. With B+ being among the most vigorous of the cultivated *P. cubensis* genotypes, mycelia appears to have developed rhizomorphic strands to overtake the bacterial colonies. Rhizomorphs represent complex aggregations of differentiated hyphal tissue with atypically dominant growth [48], which can act as conduits to enhance aerobic activity [1]. The formation of these specialized tissues was not characteristic of the other genotypes in response to contamination. On the contrary, PA halted growth toward the bacterial interface and showed growth in the opposite direction. Similarly, JMF mycelia began to thicken toward the edge opposite the contaminant. It was not possible to verify that the bacteria were the same strain in all instances of contamination, so it is difficult to directly compare the responses of mycelia to contaminants. However, since PA and JMF mycelia are characterized as being less vigorous than B+, the stark contrast in response is no surprise. Akinyele et al. [49] found that the presence of *Pseudomonas tolaasii* inhibited the growth of *Pleurotus* mycelia on semi-solid media and in liquid cultures, perhaps due to the production of volatile organic compounds (VOCs). However, VOCs have also been suggested to promote the growth of some Basidiomycete mycelia, and bacteria-borne antifungal polyketides and polypeptides have been proposed as the cause of mycelium growth inhibition in these cases [46]. Although certain bacterial strains might support mycelium growth, co-culturing psilocybin-producing mushrooms with bacteria would likely not be in line with GACP and GMP guidelines. Thus, it is recommended that any contaminated mycelium cultures be eliminated or efforts taken to remove the contaminant such as the use of antibiotic-amended media.

The set of experiments in the current work has produced valuable results that lay the foundation for the development and optimization of mycelium proliferation practices for various psilocybin-producing mushroom isolates. However, since the donor material for Experimental Series 1 was maintained on ¼ PDA and on WA for Experimental Series 2, there can be no direct comparison between these two sets of experiments. Future studies should look at comparing the best treatments of each Experimental Series, perhaps by comparing different concentrations of MEA and PDA P, or a combination of these media formulations that allows the optimal growth of *P. cubensis* genotypes along with PA. Additionally, to confirm the efficacy of PDA from raw ingredients, additional potato cultivars should be tested and compared. Future studies should also include biomass production and micro-morphological features. Furthermore, since the current work only used up to four replicates, follow-up studies with additional replicates would be able to more effectively relate morphology to media. Future studies should delve deeper into the influence of media on mycelium morphology and attempt to discern reasons why PDA made from raw natural ingredients outperformed commercialized PDA formulations.

## 5. Conclusions

The current investigation is the first to look at the mycelium growth and morphology of different *Psilocybe* spp. genotypes on various semi-solid media treatments. This work lays the foundation for the development and optimization of *Psilocybe* spp. production protocols. In general, the media used for the proliferation of mycelia should be carefully considered for its suitability with the genotype of interest. However, this might not be as important when considering the cultivation of more vigorous *P. cubensis* genotypes such as B+. In general, it seems that MEA offers an advantage for the mycelium proliferation of coprophilous *Psilocybe* spp. genotypes. Alternatively, PDA-based media offer considerable benefits for the proliferation of PA, a saprotrophic, wood-rotting genotype. Furthermore, PDA-based media derived from raw ingredients supported mycelium proliferation to a greater degree than commercialized PDA powder for most of the genotypes tested. There could be additional benefit to preparing PDA using raw, unpeeled potatoes compared to using peeled potatoes, although this remains to be confirmed. Finally, although MS media represent a more synthetically produced, precisely formulated media option, it showed little value for proliferating any of the *Psilocybe* spp. genotypes tested. The results of the current work represent important preliminary information about the growth dynamics and morphological traits of *Psilocybe* spp. mycelia that can be used to direct the future optimization and standardization of methods for the maintenance and proliferation of mother mycelium cultures.

## Figures and Tables

**Table 1:** Details relating to media recipes used as treatments for Experimental Series 1 and 2.

Media	Details	Dextrose	Potato
**Potato Dextrose Agar (PDA)**	Peeled potatoes boiled for 30 min., broth used in media.	20 g/L	200 g/L
**Potato Dextrose Agar Peel (PDA P)**	Unpeeled potatoes boiled for 30 min., broth used in media.	20 g/L	200 g/L
**Potato Dextrose Agar Powder (PDA R)**	As directed by the supplier, 24 g/L was used.	0	0
**Malt Extract Agar (MEA)**	As directed by the supplier, 20 g/L was used.	0	0
**Murashige and Skoog (MS)**	As directed by the supplier, 4.33 g/L was used.	20 g/L	0
**Water Agar (WA)**	Distilled water and agar.	0	0

**Table 2:** Growth trends on day 25 in Experimental Series 1. The mean percent colonization of the Petri dish and the mean surface area are reported. Letters represent significance with 95% confidence.

Genotype	Mean % Colonization on Day 25				Mean Surface Area Colonized on Day 25 (mm^2^)			
	WA	PDA	MEA	MS	WA	PDA	MEA	MS
APE	100%	30.5%	95.8%	4.0%	5674.50 **a**	1729.91 **b**	5436.25 **a**	225.16 **c**
B+	100%	100%	100%	15.7%	5674.50 **a**	5674.50 **a**	5674.50 **a**	890.17 **b**
JMF	95.6%	55.4%	64.2%	9.2%	5426.73 **a**	2180.84 **b**	3644.23 **c**	523.29 **d**
PA	61.8%	69.7%	47.8%	14.2%	3504.34 **a**	3957.89 **a**	2711.10 **b**	802.90 **c**

**Table 3:** Percent occurrence of morphology based on different media. Morphologies include no growth (rank 0), sparse mycelium (rank 1), sparse mycelium becoming dense (rank 2), and dense mycelium (rank 3).

Genotype	% Morphology Occurrence		
	WA	PDA	MEA
**APE**	100% 1	75% 2 25% 3	50% 2 50% 3
**B+**	100% 1	75% 3 25% 2	75% 3 25% 2
**JMF**	100% 1	100% 3	100% 3
**PA**	100% 1	100% 3	100% 2

**Table 4:** Correlation analysis of mycelium morphology based on treatments. Results are based on Pearson correlation analysis and linear regression. Pearson correlation coefficient (PCC), coefficient of determination (R^2^), and *p*-value (*p*) are shown.

Media	Correlation Assessment		
	PCC	R^2^	*p*
**PDA**	0.22	0.05	0.41
**MEA**	0.28	0.08	0.29

**Table 5:** Colonization trends on day 25 of growth for each genotype of interest. The mean percent colonization of the Petri dish and the mean surface area are reported. Letters represent significance with 95% confidence.

Genotype	Mean % Colonization on Day 25				Mean Surface Area Colonized on Day 25 (mm^2^)			
	PDA P	PDA	WA	PDA R	PDA P	PDA	WA	PDA R
APE	83.7%	72.3%	95%	0%	4751.91 **a**	4102.86 **a**	5390.62 **a**	0 **b**
B+	100%	100%	97.8%	100%	5674.50 **a**	5674.50 **a**	5548.06 **a**	5674.50 **a**
JMF	59.5%	49.1%	97.5%	32.0%	3373.50 **a**	2786.94 **a**	5534.74 **b**	1817.44 **c**
PA	96.4%	95.2%	81.3%	82.5%	5468.25 **a**	5399.45 **a**	4612.11 **b**	4678.37 **b**

**Table 6:** Percent occurrence of morphology based on different media. Morphologies include no growth (rank 0), sparse mycelium (rank 1), sparse mycelium becoming dense (rank 2), and dense mycelium (rank 3).

Genotype	% Morphology Occurrence per Treatment			
	PDA P	PDA	WA	PDA R
**APE**	100% 2	100% 2	100% 1	100% 0
**B+**	100% 3	100% 3	100% 1	100% 3
**JMF**	75% 2 25% 3	67% 2 33% 3	100% 1	50% 2 50% 3
**PA**	100% 3	100% 3	100% 1	67% 3 33% 2

**Table 7:** Correlation analysis of mycelium morphology based on treatments. Results are based on Pearson correlation analysis and linear regression. Pearson correlation coefficient (PCC), coefficient of determination (R^2^), and *p*-value (*p*) are shown.

Media	Correlation Assessment		
	PCC	R^2^	*p*
**PDA P**	0.53	0.28	0.03
**PDA**	0.41	0.17	0.15
**PDA R**	0.82	0.69	0.0001

## Data Availability

The original contributions presented in this study are included in the article/Supplementary Materials. Further inquiries can be directed to the corresponding author.

## Supplementary Materials

The following supporting information can be downloaded at https://www.mdpi.com/article/10.3390/jof11060450/s1, Table S1. Results of Tukey HSD post hoc analysis for APE throughout Experimental Series 1. Table S2. Results of Tukey HSD post hoc analysis for B+ throughout Experimental Series 1. Table S3. Results of Tukey HSD post hoc analysis for JMF throughout Experimental Series 1. Table S4. Results of Tukey HSD post hoc analysis for PA throughout Experimental Series 1. Table S5. Results of Tukey HSD post hoc analysis for APE throughout Experimental Series 2. Table S6. Results of Tukey HSD post hoc analysis for B+ throughout Experimental Series 2. Table S7. Results of Tukey HSD post hoc analysis for JMF throughout Experimental Series 2. Table S8. Results of Tukey HSD post hoc analysis for PA throughout Experimental Series 2.
