# Supplementary material for: Mycelium Growth and Development of Psilocybe spp. Mother Cultures on Agar-Based Media
Source: J Fungi (Basel). 2025 Jun 13;11(6):450. doi: 10.3390/jof11060450 (PMC12194638; doi:10.3390/jof11060450)
Supplement: Supplementary file 1 [file jof-11-00450-s001.zip › jof-3648143-supplementary.pdf]

Table S1. Results of Tukey HSD post hoc analysis for APE throughout Experimental Series 1.

Day 0	diff	lwr	upr	p adj
MS-MEA	-7.105427e-15	-3.693870e-14	2.272785e-14	0.8923559
PDA-MEA	-7.105427e-15	-3.693870e-14	2.272785e-14	0.8923559
WA-MEA	-2.131628e-14	-5.114956e-14	8.516993e-15	0.2012641
PDA-MS	0.000000e+00	-2.983328e-14	2.983328e-14	1.0000000
WA-MS	-1.421085e-14	-4.404413e-14	1.562242e-14	0.5146067
WA-PDA	-1.421085e-14	-4.404413e-14	1.562242e-14	0.5146067

Day 5	diff	lwr	upr	p adj
MS-MEA	-121.47675	-593.49935	350.5459	0.8689693
PDA-MEA	-100.43700	-572.45960	371.5856	0.9198086
WA-MEA	429.58650	-42.43610	901.6091	0.0788880
PDA-MS	21.03975	-450.98285	493.0624	0.9991240
WA-MS	551.06325	79.04065	1023.0859	0.0210091
WA-PDA	530.02350	58.00090	1002.0461	0.0264923

Day 10	diff	lwr	upr	p adj
MS-MEA	-780.7762	-1551.325	-10.22705	0.0467080
PDA-MEA	-647.5060	-1418.055	123.04320	0.1113921
WA-MEA	4316.7278	3546.179	5087.27695	0.0000000
PDA-MS	133.2702	-637.279	903.81945	0.9542745
WA-MS	5097.5040	4326.955	5868.05320	0.0000000
WA-PDA	4964.2337	4193.685	5734.78295	0.0000000

Day 15	diff	lwr	upr	p adj
MS-MEA	-3875.086	-5258.0660	-2492.106	0.0000131
PDA-MEA	-3394.651	-4777.6310	-2011.671	0.0000496
WA-MEA	1023.150	-359.8302	2406.130	0.1793882
PDA-MS	480.435	-902.5452	1863.415	0.7350093
WA-MS	4898.236	3515.2555	6281.216	0.0000011
WA-PDA	4417.801	3034.8205	5800.781	0.0000033

Day 20	diff	lwr	upr	p adj
MS-MEA	-4897.8367	-6236.3359	-3559.338	0.0000008
PDA-MEA	-3836.9307	-5175.4299	-2498.432	0.0000103
WA-MEA	339.1582	-999.3409	1677.657	0.8739831
PDA-MS	1060.9060	-277.5931	2399.405	0.1401563
WA-MS	5236.9950	3898.4959	6575.494	0.0000004
WA-PDA	4176.0890	2837.5899	5514.588	0.0000043

Day 25	diff	lwr	upr	p adj
MS-MEA	-5211.0957	-6683.90979	-3738.282	0.0000011

PDA-MEA	-3706.3457	-5179.15979	-2233.532	0.0000388
WA-MEA	238.2472	-1234.56679	1711.061	0.9620302
PDA-MS	1504.7500	31.93596	2977.564	0.0447312
WA-MS	5449.3430	3976.52896	6922.157	0.0000007
WA-PDA	3944.5930	2471.77896	5417.407	0.0000207

Table S2. Results of Tukey HSD post hoc analysis for B+ throughout Experimental Series 1.

				Day 0
	diff	lwr	upr	p adj
MS-MEA	-7.105427e-15	-3.693870e-14	2.272785e-14	0.8923559
PDA-MEA	-7.105427e-15	-3.693870e-14	2.272785e-14	0.8923559
WA-MEA	-2.131628e-14	-5.114956e-14	8.516993e-15	0.2012641
PDA-MS	0.000000e+00	-2.983328e-14	2.983328e-14	1.0000000
WA-MS	-1.421085e-14	-4.404413e-14	1.562242e-14	0.5146067
WA-PDA	-1.421085e-14	-4.404413e-14	1.562242e-14	0.5146067
				Day 5
	diff	lwr	upr	p adj
MS-MEA	-545.4542	-708.93851	-381.9700	0.0000021
PDA-MEA	-37.1145	-200.59876	126.3698	0.9049182
WA-MEA	142.3587	-21.12551	305.8430	0.0959294
PDA-MS	508.3397	344.85549	671.8240	0.0000044
WA-MS	687.8130	524.32874	851.2973	0.0000002
WA-PDA	179.4733	15.98899	342.9575	0.0301780
				Day 10
	diff	lwr	upr	p adj
MS-MEA	-3896.5312	-4701.043	-3092.01997	0.0000000
PDA-MEA	-429.3633	-1233.875	375.14803	0.4224616
WA-MEA	-1159.3760	-1963.887	-354.86472	0.0051078
PDA-MS	3467.1680	2662.657	4271.67928	0.0000001
WA-MS	2737.1552	1932.644	3541.66653	0.0000017
WA-PDA	-730.0127	-1534.524	74.49853	0.0799610
				Day 15
	diff	lwr	upr	p adj
MS-MEA	-5014.288	-5418.0462	-4610.5308	0.0000000
PDA-MEA	-74.449	-478.2067	329.3087	0.9454754
WA-MEA	-1154.467	-1558.2247	-750.7093	0.0000106
PDA-MS	4939.839	4536.0818	5343.5972	0.0000000
WA-MS	3859.821	3456.0638	4263.5792	0.0000000
WA-PDA	-1080.018	-1483.7757	-676.2603	0.0000210
				Day 20
	diff	lwr	upr	p adj
MS-MEA	-4901.0257	-5294.9262	-4507.1253	0.0000000
PDA-MEA	0.0000	-393.9005	393.9005	1.0000000

	diff	lwr	upr	p adj
WA-MEA	-240.9225	-634.8230	152.9780	0.3129925
PDA-MS	4901.0257	4507.1253	5294.9262	0.0000000
WA-MS	4660.1032	4266.2028	5054.0037	0.0000000
WA-PDA	-240.9225	-634.8230	152.9780	0.3129925

Day 25

	diff	lwr	upr	p adj
MS-MEA	-4.784330e+03	-5084.9317	-4483.7283	0
PDA-MEA	0.000000e+00	-300.6017	300.6017	1
WA-MEA	9.094947e-13	-300.6017	300.6017	1
PDA-MS	4.784330e+03	4483.7283	5084.9317	0
WA-MS	4.784330e+03	4483.7283	5084.9317	0
WA-PDA	9.094947e-13	-300.6017	300.6017	1

Table S3. Results of Tukey HSD post hoc analysis for JMF throughout Experimental Series 1.

Day 0

	diff	lwr	upr	p adj
MS-MEA	-7.105427e-15	-3.693870e-14	2.272785e-14	0.8923559
PDA-MEA	-7.105427e-15	-3.693870e-14	2.272785e-14	0.8923559
WA-MEA	-2.131628e-14	-5.114956e-14	8.516993e-15	0.2012641
PDA-MS	0.000000e+00	-2.983328e-14	2.983328e-14	1.0000000
WA-MS	-1.421085e-14	-4.404413e-14	1.562242e-14	0.5146067
WA-PDA	-1.421085e-14	-4.404413e-14	1.562242e-14	0.5146067

Day 5

	diff	lwr	upr	p adj
MS-MEA	-145.75200	-225.44637	-66.05763	0.0007593
PDA-MEA	-156.12475	-235.81912	-76.43038	0.0004151
WA-MEA	484.48275	404.78838	564.17712	0.0000000
PDA-MS	-10.37275	-90.06712	69.32162	0.9794783
WA-MS	630.23475	550.54038	709.92912	0.0000000
WA-PDA	640.60750	560.91313	720.30187	0.0000000

Day 10

	diff	lwr	upr	p adj
MS-MEA	-982.9735	-1891.0033	-74.94372	0.0326651
PDA-MEA	-921.1855	-1829.2153	-13.15572	0.0464171
WA-MEA	793.8575	-114.1723	1701.88728	0.0942920
PDA-MS	61.7880	-846.2418	969.81778	0.9969172
WA-MS	1776.8310	868.8012	2684.86078	0.0004194
WA-PDA	1715.0430	807.0132	2623.07278	0.0005738

Day 15

	diff	lwr	upr	p adj
MS-MEA	-1412.9012	-2189.9904	-635.8121	0.0007986
PDA-MEA	-1174.2950	-1951.3841	-397.2059	0.0035817

	diff	lwr	upr	p adj
WA-MEA	2882.8565	2105.7674	3659.9456	0.0000007
PDA-MS	238.6063	-538.4829	1015.6954	0.7991858
WA-MS	4295.7577	3518.6686	5072.8469	0.0000000
WA-PDA	4057.1515	3280.0624	4834.2406	0.0000000

Day 20

	diff	lwr	upr	p adj
MS-MEA	-2330.2202	-3130.5838	-1529.8567	0.0000088
PDA-MEA	-1737.7460	-2538.1095	-937.3825	0.0001616
WA-MEA	2343.5633	1543.1997	3143.9268	0.0000083
PDA-MS	592.4743	-207.8893	1392.8378	0.1790252
WA-MS	4673.7835	3873.4200	5474.1470	0.0000000
WA-PDA	4081.3092	3280.9457	4881.6728	0.0000000

Day 25

	diff	lwr	upr	p adj
MS-MEA	-3120.939	-4074.9003	-2166.9787	0.0000026
PDA-MEA	-1463.391	-2417.3516	-509.4299	0.0031927
WA-MEA	1782.500	828.5394	2736.4611	0.0006305
PDA-MS	1657.549	703.5879	2611.5096	0.0011733
WA-MS	4903.440	3949.4789	5857.4006	0.0000000
WA-PDA	3245.891	2291.9302	4199.8518	0.0000017

Table S4. Results of Tukey HSD post hoc analysis for PA throughout Experimental Series 1.

Day 0

	diff	lwr	upr	p adj
MS-MEA	-7.105427e-15	-3.693870e-14	2.272785e-14	0.8923559
PDA-MEA	-7.105427e-15	-3.693870e-14	2.272785e-14	0.8923559
WA-MEA	-2.131628e-14	-5.114956e-14	8.516993e-15	0.2012641
PDA-MS	0.000000e+00	-2.983328e-14	2.983328e-14	1.0000000
WA-MS	-1.421085e-14	-4.404413e-14	1.562242e-14	0.5146067
WA-PDA	-1.421085e-14	-4.404413e-14	1.562242e-14	0.5146067

Day 5

	diff	lwr	upr	p adj
MS-MEA	-98.95675	-143.54214	-54.371356	0.0001313
PDA-MEA	-36.00825	-80.59364	8.577144	0.1304627
WA-MEA	20.01875	-24.56664	64.604144	0.5609281
PDA-MS	62.94850	18.36311	107.533894	0.0059298
WA-MS	118.97550	74.39011	163.560894	0.0000215
WA-PDA	56.02700	11.44161	100.612394	0.0132111

Day 10

	diff	lwr	upr	p adj
MS-MEA	-302.7707	-477.791178	-127.7503	0.0012172
PDA-MEA	-19.0430	-194.063428	155.9774	0.9877621
WA-MEA	162.7015	-12.318928	337.7219	0.0715192
PDA-MS	283.7277	108.707322	458.7482	0.0020697

	diff	lwr	upr	p adj
WA-MS	465.4722	290.451822	640.4927	0.0000222
WA-PDA	181.7445	6.724072	356.7649	0.0410365

Day 15				
	diff	lwr	upr	p adj
MS-MEA	-517.0730	-873.033519	-161.1125	0.0048169
PDA-MEA	426.8228	70.862231	782.7833	0.0178205
WA-MEA	784.4725	428.511981	1140.4330	0.0001405
PDA-MS	943.8957	587.935231	1299.8563	0.0000229
WA-MS	1301.5455	945.584981	1657.5060	0.0000008
WA-PDA	357.6497	1.689231	713.6103	0.0487980

Day 20				
	diff	lwr	upr	p adj
MS-MEA	-1053.55325	-1567.1276	-539.9789	0.0002736
PDA-MEA	932.23775	418.6634	1445.8121	0.0008100
WA-MEA	943.62500	430.0507	1457.1993	0.0007296
PDA-MS	1985.79100	1472.2167	2499.3653	0.0000004
WA-MS	1997.17825	1483.6039	2510.7526	0.0000004
WA-PDA	11.38725	-502.1871	524.9616	0.9998915

Day 25				
	diff	lwr	upr	p adj
MS-MEA	-1908.2072	-2478.5406	-1337.8739	0.0000020
PDA-MEA	1246.7905	676.4571	1817.1239	0.0001516
WA-MEA	793.2400	222.9066	1363.5734	0.0066042
PDA-MS	3154.9977	2584.6644	3725.3311	0.0000000
WA-MS	2701.4472	2131.1139	3271.7806	0.0000000
WA-PDA	-453.5505	-1023.8839	116.7829	0.1384147

Table S5. Results of Tukey HSD post hoc analysis for APE throughout Experimental Series 2.

Day 0				
	diff	lwr	upr	p adj
PDA Peel-PDA	-2.131628e-14	-5.114956e-14	8.516993e-15	0.2012641
PDA Powder-PDA	0.000000e+00	-2.983328e-14	2.983328e-14	1.0000000
WA-PDA	-7.105427e-15	-3.693870e-14	2.272785e-14	0.8923559
PDA Powder-PDA Peel	2.131628e-14	-8.516993e-15	5.114956e-14	0.2012641
WA-PDA Peel	1.421085e-14	-1.562242e-14	4.404413e-14	0.5146067
WA-PDA Powder	-7.105427e-15	-3.693870e-14	2.272785e-14	0.8923559

Day 5				
	diff	lwr	upr	p adj
PDA Peel-PDA	62.16575	-334.95367	459.2852	0.9653617
PDA Powder-PDA	-80.28675	-477.40617	316.8327	0.9300321
WA-PDA	290.37000	-106.74942	687.4894	0.1866110
PDA Powder-PDA Peel	-142.45250	-539.57192	254.6669	0.7161532
WA-PDA Peel	228.20425	-168.91517	625.3237	0.3623858

WA-PDA Powder	370.65675	-26.46267	767.7762	0.0701799

Day 10				
	diff	lwr	upr	p adj
PDA Peel-PDA	198.0352	-999.978355	1396.0489	0.9596682
PDA Powder-PDA	-506.4388	-1704.452355	691.5749	0.6062131
WA-PDA	1393.6000	195.586395	2591.6136	0.0214725
PDA Powder-PDA Peel	-704.4740	-1902.487605	493.5396	0.3439550
WA-PDA Peel	1195.5647	-2.448855	2393.5784	0.0505265
WA-PDA Powder	1900.0387	702.025145	3098.0524	0.0024629

Day 15				
	diff	lwr	upr	p adj
PDA Peel-PDA	417.725	-1089.880	1925.3304	0.8427439
PDA Powder-PDA	-1464.762	-2972.367	42.8439	0.0578157
WA-PDA	3086.100	1578.495	4593.7054	0.0002790
PDA Powder-PDA Peel	-1882.486	-3390.092	-374.8811	0.0137691
WA-PDA Peel	2668.375	1160.770	4175.9804	0.0010044
WA-PDA Powder	4550.861	3043.256	6058.4669	0.0000060

Day 20				
	diff	lwr	upr	p adj
PDA Peel-PDA	411.162	-2125.9190	2948.2430	0.9618347
PDA Powder-PDA	-3018.979	-5556.0595	-481.8975	0.0186879
WA-PDA	2128.051	-409.0303	4665.1318	0.1122299
PDA Powder-PDA Peel	-3430.140	-5967.2215	-893.0595	0.0080637
WA-PDA Peel	1716.889	-820.1923	4253.9698	0.2379534
WA-PDA Powder	5147.029	2609.9482	7684.1103	0.0003028

Day 25				
	diff	lwr	upr	p adj
PDA Peel-PDA	649.0563	-1174.3409	2472.453	0.7207667
PDA Powder-PDA	-4052.5855	-5875.9826	-2229.188	0.0001296
WA-PDA	1287.7625	-535.6346	3111.160	0.2088736
PDA Powder-PDA Peel	-4701.6418	-6525.0389	-2878.245	0.0000304
WA-PDA Peel	638.7062	-1184.6909	2462.103	0.7302207
WA-PDA Powder	5340.3480	3516.9509	7163.745	0.0000083

Table S6. Results of Tukey HSD post hoc analysis for B+ throughout Experimental Series 2.

Day 0				
	diff	lwr	upr	p adj
PDA Peel-PDA	-2.131628e-14	-5.114956e-14	8.516993e-15	0.2012641
PDA Powder-PDA	0.000000e+00	-2.983328e-14	2.983328e-14	1.0000000
WA-PDA	-7.105427e-15	-3.693870e-14	2.272785e-14	0.8923559
PDA Powder-PDA Peel	2.131628e-14	-8.516993e-15	5.114956e-14	0.2012641
WA-PDA Peel	1.421085e-14	-1.562242e-14	4.404413e-14	0.5146067
WA-PDA Powder	-7.105427e-15	-3.693870e-14	2.272785e-14	0.8923559

Day 5

	diff	lwr	upr	p adj
PDA Peel-PDA	-139.3462	-361.85520	83.16287	0.2885412
PDA Powder-PDA	-187.1067	-409.61570	35.40237	0.1094052
WA-PDA	101.0666	-121.44245	323.57562	0.5431864
PDA Powder-PDA Peel	-47.7605	-253.76384	158.24284	0.8958278
WA-PDA Peel	240.4128	34.40941	446.41609	0.0215770
WA-PDA Powder	288.1733	82.16991	494.17659	0.0067951

Day 10

	diff	lwr	upr	p adj
PDA Peel-PDA	-130.4642	-1013.0008	752.0725	0.9692584
PDA Powder-PDA	-674.6607	-1557.1973	207.8760	0.1569786
WA-PDA	-336.2852	-1218.8218	546.2515	0.6700604
PDA Powder-PDA Peel	-544.1965	-1361.2667	272.8737	0.2439162
WA-PDA Peel	-205.8210	-1022.8912	611.2492	0.8713823
WA-PDA Powder	338.3755	-478.6947	1155.4457	0.6124748

Day 15

	diff	lwr	upr	p adj
PDA Peel-PDA	109.7367	-966.1044	1185.5777	0.9894029
PDA Powder-PDA	-400.5458	-1476.3869	675.2952	0.6851418
WA-PDA	-672.2131	-1748.0541	403.6279	0.2902447
PDA Powder-PDA Peel	-510.2825	-1506.3177	485.7527	0.4473888
WA-PDA Peel	-781.9497	-1777.9850	214.0855	0.1426131
WA-PDA Powder	-271.6673	-1267.7025	724.3680	0.8434935

Day 20

	diff	lwr	upr	p adj
PDA Peel-PDA	-9.094947e-13	-557.6375	557.6375	1.0000000
PDA Powder-PDA	-1.326157e+02	-690.2532	425.0217	0.8888055
WA-PDA	-3.568538e+02	-914.4912	200.7837	0.2725400
PDA Powder-PDA Peel	-1.326157e+02	-648.8877	383.6562	0.8649529
WA-PDA Peel	-3.568537e+02	-873.1257	159.4182	0.2185942
WA-PDA Powder	-2.242380e+02	-740.5100	292.0340	0.5773581

Day 25

	diff	lwr	upr	p adj
PDA Peel-PDA	-9.094947e-13	-185.3720	185.37200	1.000000
PDA Powder-PDA	0.000000e+00	-185.3720	185.37200	1.000000
WA-PDA	-1.264447e+02	-311.8167	58.92725	0.227489
PDA Powder-PDA Peel	9.094947e-13	-171.6211	171.62112	1.000000
WA-PDA Peel	-1.264447e+02	-298.0659	45.17637	0.178260
WA-PDA Powder	-1.264447e+02	-298.0659	45.17637	0.178260

Table S7. Results of Tukey HSD post hoc analysis for JMF throughout Experimental Series 2.

Day 0

	diff	lwr	upr	p adj
PDA Peel-PDA	-2.131628e-14	-5.114956e-14	8.516993e-15	0.2012641
PDA Powder-PDA	0.000000e+00	-2.983328e-14	2.983328e-14	1.0000000
WA-PDA	-7.105427e-15	-3.693870e-14	2.272785e-14	0.8923559
PDA Powder-PDA Peel	2.131628e-14	-8.516993e-15	5.114956e-14	0.2012641
WA-PDA Peel	1.421085e-14	-1.562242e-14	4.404413e-14	0.5146067
WA-PDA Powder	-7.105427e-15	-3.693870e-14	2.272785e-14	0.8923559

Day 5

	diff	lwr	upr	p adj
PDA Peel-PDA	-37.96475	-165.3130	89.38350	0.8125937
PDA Powder-PDA	-77.48450	-204.8328	49.86375	0.3170588
WA-PDA	342.11475	214.7665	469.46300	0.0000201
PDA Powder-PDA Peel	-39.51975	-166.8680	87.82850	0.7941854
WA-PDA Peel	380.07950	252.7312	507.42775	0.0000068
WA-PDA Powder	419.59925	292.2510	546.94750	0.0000024

Day 10

	diff	lwr	upr	p adj
PDA Peel-PDA	-52.11683	-419.2117	314.9780	0.9725762
PDA Powder-PDA	-188.19833	-555.2932	178.8965	0.4468272
WA-PDA	1896.16442	1529.0696	2263.2592	0.0000000
PDA Powder-PDA Peel	-136.08150	-475.9453	203.7823	0.6364143
WA-PDA Peel	1948.28125	1608.4175	2288.1450	0.0000000
WA-PDA Powder	2084.36275	1744.4990	2424.2265	0.0000000

Day 15

	diff	lwr	upr	p adj
PDA Peel-PDA	-8.47125	-298.8808	281.9383	0.9997418
PDA Powder-PDA	-401.58200	-691.9915	-111.1725	0.0073533
WA-PDA	3441.97800	3151.5685	3732.3875	0.0000000
PDA Powder-PDA Peel	-393.11075	-661.9777	-124.2438	0.0049912
WA-PDA Peel	3450.44925	3181.5823	3719.3162	0.0000000
WA-PDA Powder	3843.56000	3574.6930	4112.4270	0.0000000

Day 20

	diff	lwr	upr	p adj
PDA Peel-PDA	241.8684	-587.7561	1071.49292	0.8164182
PDA Powder-PDA	-595.2781	-1424.9026	234.34642	0.1944600
WA-PDA	3371.3377	2541.7132	4200.96217	0.0000005
PDA Powder-PDA Peel	-837.1465	-1605.2295	-69.06346	0.0318242
WA-PDA Peel	3129.4693	2361.3862	3897.55229	0.0000005
WA-PDA Powder	3966.6158	3198.5327	4734.69879	0.0000000

Day 25

	diff	lwr	upr	p adj
PDA Peel-PDA	586.5514	-172.725	1345.8278	0.1512166
PDA Powder-PDA	-969.5068	-1728.783	-210.2304	0.0124340
WA-PDA	2747.7939	1988.517	3507.0703	0.0000016

	diff	lwr	upr	p adj
PDA Powder-PDA Peel	-1556.0583	-2259.012	-853.1049	0.0001774
WA-PDA Peel	2161.2425	1458.289	2864.1959	0.0000082
WA-PDA Powder	3717.3008	3014.347	4420.2541	0.0000000

Table S8. Results of Tukey HSD post hoc analysis for PA throughout Experimental Series 2.

				Day 0
	diff	lwr	upr	p adj
PDA Peel-PDA	-2.131628e-14	-5.114956e-14	8.516993e-15	0.2012641
PDA Powder-PDA	0.000000e+00	-2.983328e-14	2.983328e-14	1.0000000
WA-PDA	-7.105427e-15	-3.693870e-14	2.272785e-14	0.8923559
PDA Powder-PDA Peel	2.131628e-14	-8.516993e-15	5.114956e-14	0.2012641
WA-PDA Peel	1.421085e-14	-1.562242e-14	4.404413e-14	0.5146067
WA-PDA Powder	-7.105427e-15	-3.693870e-14	2.272785e-14	0.8923559
				Day 5
	diff	lwr	upr	p adj
PDA Peel-PDA	13.71375	-71.4631516	98.89065	0.9625265
PDA Powder-PDA	-20.00650	-105.1834016	65.17040	0.8961432
WA-PDA	64.17400	-21.0029016	149.35090	0.1684738
PDA Powder-PDA Peel	-33.72025	-118.8971516	51.45665	0.6527047
WA-PDA Peel	50.46025	-34.7166516	135.63715	0.3380567
WA-PDA Powder	84.18050	-0.9964016	169.35740	0.0530859
				Day 10
	diff	lwr	upr	p adj
PDA Peel-PDA	-113.4935	-230.153367	3.166367	0.0574408
PDA Powder-PDA	-353.1790	-469.838867	-236.519133	0.0000058
WA-PDA	-5.9040	-122.563867	110.755867	0.9987209
PDA Powder-PDA Peel	-239.6855	-356.345367	-123.025633	0.0002698
WA-PDA Peel	107.5895	-9.070367	224.249367	0.0742205
WA-PDA Powder	347.2750	230.615133	463.934867	0.0000070
				Day 15
	diff	lwr	upr	p adj
PDA Peel-PDA	-95.6985	-409.21001	217.8130	0.8019205
PDA Powder-PDA	-786.9827	-1100.49426	-473.4712	0.0000397
WA-PDA	-555.1665	-868.67801	-241.6550	0.0010003
PDA Powder-PDA Peel	-691.2843	-1004.79576	-377.7727	0.0001398
WA-PDA Peel	-459.4680	-772.97951	-145.9565	0.0045104
WA-PDA Powder	231.8163	-81.69526	545.3278	0.1797152
				Day 20
	diff	lwr	upr	p adj
PDA Peel-PDA	242.37100	-176.0852	660.8272	0.3487185
PDA Powder-PDA	-923.89375	-1375.8781	-471.9094	0.0003560
WA-PDA	-918.70200	-1337.1582	-500.2458	0.0001909
PDA Powder-PDA Peel	-1166.26475	-1618.2491	-714.2804	0.0000438

WA-PDA Peel	-1161.07300	-1579.5292	-742.6168	0.0000220
WA-PDA Powder	5.19175	-446.7926	457.1761	0.9999842

Day 25

	diff	lwr	upr	p adj
PDA Peel-PDA	68.80875	-690.9358	828.55333	0.9925118
PDA Powder-PDA	-721.07342	-1541.6914	99.54452	0.0911127
WA-PDA	-787.33575	-1547.0803	-27.59117	0.0416765
PDA Powder-PDA Peel	-789.88217	-1610.5001	30.73577	0.0602744
WA-PDA Peel	-856.14450	-1615.8891	-96.39992	0.0264146
WA-PDA Powder	-66.26233	-886.8803	754.35561	0.9946550
